# Use and Awareness of The Community Guide in State and Local Health Department Chronic Disease Programs

**DOI:** 10.5888/pcd17.200196

**Published:** 2020-10-22

**Authors:** Emily Rodriguez Weno, Stephanie Mazzucca, Renee G. Parks, Margaret Padek, Peg Allen, Ross C. Brownson

**Affiliations:** 1 Prevention Research Center, Brown School, Washington University in St. Louis, St. Louis, Missouri; 2 Department of Surgery, Division of Public Health Sciences, Alvin J. Siteman Cancer Center, Washington University School of Medicine, Washington University in St. Louis, St. Louis, Missouri

## Abstract

**Introduction:**

The Community Guide (Guide) is a user-friendly, systematic review system that provides information on evidence-based interventions (EBIs) in public health practice. Little is known about what predicts Guide awareness and use in state health departments (SHDs) and local health departments (LHDs).

**Methods:**

We pooled data from 3 surveys (administered in 2016, 2017, and 2018) to employees in chronic disease programs at SHDs and LHDs. Participants (n = 1,039) represented all 50 states. The surveys asked about department practices and individual, organizational, and external factors related to decisions about EBIs. We used χ^2^ tests of independence for analyses.

**Results:**

Eighty-one percent (n = 498) of SHD and 54% (n = 198) of LHD respondents reported their agency uses the Guide. Additionally, 13% of SHD participants reported not being aware of the Guide. Significant relationships were found between reporting using the Guide and academic collaboration, population size, rated importance of forming partnerships, and accreditation.

**Conclusion:**

Awareness and use of the Guide in LHD and SHD chronic disease programs is widespread. Awareness of the Guide can be vital to implementation practice, because it enhances implementation of EBI practices. However, awareness of the Guide alone is likely not enough for health departments to implement EBIs. Changes at the organizational level, including sharing information about the Guide and providing training on how to best use it, may increase its awareness and use.

**MEDSCAPE CME**

In support of improving patient care, this activity has been planned and implemented by Medscape, LLC and *Preventing Chronic Disease*. Medscape, LLC is jointly accredited by the Accreditation Council for Continuing Medical Education (ACCME), the Accreditation Council for Pharmacy Education (ACPE), and the American Nurses Credentialing Center (ANCC), to provide continuing education for the healthcare team.

Medscape, LLC designates this Journal-based CME activity for a maximum of 1.00 AMA PRA Category 1 Credit(s)™. Physicians should claim only the credit commensurate with the extent of their participation in the activity.

Successful completion of this CME activity, which includes participation in the evaluation component, enables the participant to earn up to 1.0 MOC points in the American Board of Internal Medicine’s (ABIM) Maintenance of Certification (MOC) program. Participants will earn MOC points equivalent to the amount of CME credits claimed for the activity. It is the CME activity provider’s responsibility to submit participant completion information to ACCME for the purpose of granting ABIM MOC credit.

**Release date: October 22, 2020; Expiration date: October 22, 2021**

**Learning Objectives**

Upon completion of this activity, participants will be able to:

1. Describe prevalence of and characteristics related to use and awareness of the Guide by LHDs, according to a pooled analysis of self-report data from recent national surveys
2. Determine prevalence of and characteristics related to use and awareness of the Guide by SHDs, according to a pooled analysis of self-report data from recent national surveys
3. Analyze Identify pubic heath implications of prevalence of and characteristics related to use and awareness of the Guide by LHDs and SHDs, according to a poled analysis of self-report data from recent national surveys

EDITOR

Camille Martin, RD

Editor

Preventing Chronic Disease

Disclosure: Camille Martin has disclosed no relevant financial relationships.

AUTHORS

Emily Rodriguez Weno, BA

Prevention Research Center in St Louis

Brown School at Washington University in St Louis

Disclosure: Emily Rodriguez Weno, BA, has disclosed the following relevant financial relationships:

Employed by commercial interest: Bayer HealthCare Pharmaceuticals

Stephanie Mazzucca, PhD

Prevention Research Center in St Louis

Brown School at Washington University in St Louis

Disclosure: Stephanie Mazzucca, PhD, has disclosed no relevant financial relationships.

Renee G. Parks, MS

Prevention Research Center in St Louis

Washington University in St Louis

Disclosure: Renee G. Parks, MS, has disclosed no relevant financial relationships.

Margaret Padek, MPH, MSW

Prevention Research Center in St Louis

Brown School at Washington University in St Louis

Disclosure: Margaret Padek, MPH, MSW, has disclosed no relevant financial relationships.

Peg Allen, MPH, PhD, RN

Prevention Research Center in St Louis

Brown School at Washington University in St Louis

Disclosure: Peg Allen, MPH, PhD, RN, has disclosed no relevant financial relationships.

Ross C. Brownson, PhD

Prevention Research Center in St Louis

Brown School at Washington University in St Louis

St Louis University School of Medicine

Department of Surgery

Alvin J. Siteman Cancer Center

St Louis, Missouri

Disclosure: Ross C. Brownson, PhD, has disclosed no relevant financial relationships.

CME AUTHOR

Laurie Barclay, MD

Freelance writer and reviewer

Medscape, LLC

Disclosure: Laurie Barclay, MD, has disclosed no relevant financial relationships.

**Summary**

**What is already known on this topic?**

Little is known about the prevalence of and characteristics related to the awareness and use of The Community Guide (Guide) in public health departments.

**What is added by this report?**

This report fills a gap in knowledge about prevalence of use and awareness of the Guide in state and local health departments as well as related characteristics.

**What are the implications for public health practice?**

Practitioners in local health departments used the Guide less than practitioners in state health departments did. Opportunity exists to increase the use of the Guide and potentially increase implementation of evidence-based interventions with its promotion.

## Introduction

Chronic diseases account for most death and disability in the United States (1). Evidence-based interventions (EBIs) are available to prevent or lessen disease burden. Using interventions supported by evidence improves likelihood of program success, increases productivity, is ethical, and encourages efficient use of resources (2).

The more than 3,000 local health departments (LHDs) and 50 state health departments (SHDs) in the United States are well placed to implement EBIs for chronic disease prevention. However, public health practitioners reported that only 58% of previous programs were evidence-based (3). Health departments have experienced multiple challenges in implementing EBIs (4) and vary widely in the use of EBIs (5), especially those requiring clinical collaborations (6) or policy and environmental changes (7). The availability of scientific information about effective policies and programs is one of the first steps to making public health practice and agencies more evidence-based (8). One resource for this information is The Community Guide (Guide).

The Guide is a free online resource that summarizes systematic review evidence and provides recommendations on effective community preventive services, programs, and policies. It includes recommendations from a Centers for Disease Control and Prevention (CDC)–appointed task force of health experts on more than 230 interventions in 21 topics areas (9). The Guide is intended to be a tool used by public health professionals, health care providers, researchers, and decision makers in state and local communities to improve community health. The Guide is a tool that agencies can use to become more evidence-based in their practice.

The aim of this pooled analysis was to better understand characteristics related to the use and awareness of the Guide to inform strategies to increase the use of the Guide in LHDs and SHDs. The aim was accomplished by examining the prevalence of, use of, and awareness of the Guide among US public health practitioners and related factors, using self-reported data from 3 recent national surveys.

## Methods

For this cross-sectional study, we used data from 2 national surveys of employees of SHDs and LHDs, as well as data from the 2016 National Association of County and City Health Officials (NACCHO) National Profile of Local Health Departments survey (NACCHO Profile).

### 2017 Local health department survey

In 2017, we administered an online survey to the lead employee for chronic disease control at LHDs in the United States. This project was approved by Washington University in St. Louis’ institutional review board. The 86-item survey included questions about agency and individual characteristics, use of EBIs, and skills related to and organizational supports for evidence-based decision making. More information about the development of the survey instrument can be found elsewhere (10).

We identified a stratified sample of 600 eligible LHDs based on jurisdiction population size, 200 LHDs from each population size category: small (<50,000 residents), medium (50,000–200,000 residents) and large (>200,000 residents). LHDs were eligible if they reported implementation of either diabetes or body mass index screening, or population-based nutrition or physical activity efforts in the 2016 NACCHO National Profile survey (11). The employee who led chronic disease control for each LHD was identified and invited to participate, where possible, or the LHD director was invited. After excluding invalid email addresses, 579 LHDs were invited, and 376 completed surveys were included in analyses.

Participants were sent the survey link (Qualtrics) via email and received subsequent correspondence to boost responses (up to 3 emails and 2 telephone calls). Participants were offered a $20 Amazon gift card upon completing the survey. More details on the sampling methods and data collection are reported elsewhere (10).

### NACCHO 2016 National Profile of Local Health Departments

The NACCHO Profile is a survey conducted to comprehensively describe LHD infrastructure and practice (11). Details of the survey process are described elsewhere (11). The 2016 Profile included responses from 1,890 LHDs; our analysis included data from the 376 LHDs that also participated in the 2017 LHD survey. Whether the same individual responded to both the 2016 Profile and 2017 LHD survey is unknown.

### 2018 State health department survey

In 2018, we administered an online survey to individuals working in SHD chronic disease programs in all 50 US states. This project was approved by Washington University in St. Louis’ institutional review board. The 86-item survey was developed on the basis of previous work by our team and a review of the literature. Survey questions were categorized into individual, organizational, and external factors. Survey drafts for each domain were updated, mapped into the 5 domains of Administrative Evidence-Based Practice, which are organizational supports for evidence-based decision making, and underwent 3 separate reviews with team members and an advisory committee to develop a draft of the study instrument (12). The survey draft was modified based on cognitive response testing. Test–retest showed adequate reliability.

Eligible participants were drawn from the National Association of Chronic Disease Directors membership list. US territories were excluded from the survey. We selected a random sample of 1,329 individuals to invite to participate in our survey. Survey administration and recruitment was the same as for the 2017 LHD survey. We offered participants a $20 Amazon gift card for completing the survey or the option to have us donate their incentive to a public health charity.

### Measures

Both LHD and SHD surveys asked about department practices and individual, organizational, and external factors related to decisions about EBIs using Likert scale items. The main outcome variable for this analysis was reported use of the Guide. The SHD survey asked about use of the Guide; however, the LHD survey did not have a question specifically about the Guide. To examine factors related to the use of the Guide in LHDs, the question about use of the Guide from the 2016 NACCHO Profile was combined with the 2017 LHD survey variables. Correlate variables were used from the LHD and SHD surveys to quantify relationships between use of the Guide and other individual, organizational, and external factors.

### Analysis

We conducted descriptive analyses to summarize participants in both the LHD and SHD surveys. The main goal of the inferential analyses was to examine the relationships between different organizational-level factors of health departments and use and awareness of the Guide. Dichotomized variables were created from survey items using a Likert scale. Depending on the item, the Likert scale was “strongly disagree” to “agree” or “never” to “always.” SHD items with a 5-point scale were dichotomized by coding responses 1 through 3 as “disagree” and responses 4 and 5 “agree.” LHD items using a 7-point scale were dichotomized by coding responses 6 and 7 as “agree or strongly agree” and responses 1 through 5 as all else. The SHD question about participant’s work-unit use of the Guide was dichotomized with “yes, sometimes” and “yes, often” recoded as “use the Guide,” and responses of no and “I am not familiar” were coded as “do not use the Guide.” For SHDs, “I am not familiar” was recoded as “not aware of the Guide” and the 3 other response options were recoded as “aware of the Guide.” For LHDs, the NACCHO Profile asked to what extent the LHD used the Guide over the previous 12 months. We created a dichotomized variable where “LHD staff in some programmatic areas have used the Community Guide” and “LHD staff consistently use the Community Guide in all relevant programmatic areas” were coded as “use the Guide*”* and “do not know the extent of use of Community Guide within LHD” and “LHD staff have not used the Community Guide” were coded as “do not use the Guide.”

We used χ^2^ tests of independence to determine significant differences in distributions of factors and use or awareness of the Guide. Analyses were performed in SPSS version 26 (IBM Corporation), and significance was set at *P *<.05. LHD data are from the 2017 survey conducted by our study team unless noted otherwise.

## Results

The SHD survey had a 50% response rate (n = 663 of 1,329), and the LHD survey had a 65% (n = 376 of 579 ) response rate (Table 1). Most participants in both surveys were women. In SHDs, 37% of participants had a formal public health education, and in LHDs, 31% of participants did. The average number of years worked in public health was 14.8 (mean, 9.4) years for SHD participants and 16.4 (mean, 9.7) years for LHD participants. In SHDs, 14% of participants were directors of multiple programs, 51% were program managers, and 33% were specialists (eg, epidemiologists, nurses). In LHDs, 46% of participants were directors of multiple programs, 45% were program managers, and 6% were specialists.

**Table 1:** Demographic Characteristics of State Health Department and Local Health Department Participants (N = 1,039), Study on Use and Awareness of The Community Guide, United States, 2016–2018^a^

Characteristic	SHD Survey Participants (n = 663)	LHD Survey Participants (n = 376)
**Population size**		
Small (SHD <2.5 million, LHD <50,000)	181 (27)	119 (32)
Medium (SHD 2.5–6.4 million, LHD 50,000–200,000)	225 (34)	129 (34)
Large (SHD >6.4 million, LHD >200,000)	233 (35)	128 (34)
**Region^b^**		
New England	106 (17)	38 (10)
South	147 (23)	102 (27)
West	104 (16)	37 (10)
Mountains/Midwest	149 (23)	67 (18)
Mid-Atlantic and Great Lakes	137 (21)	132 (35)
**Program area^c^**		
Obesity	80 (12)	0
Tobacco	68 (11)	10 (3)
Cancer	91 (14)	—
Diabetes	38 (6)	4 (1)
Cardiovascular disease	45 (7)	—
Physical activity or nutrition	—	4 (1)
Multiple areas	172 (27)	319 (95)
Other areas	149 (23)	—
**Sex**		
Female	528 (80**)**	312 (83)
Other gender identity	3 (1)	0
Male	131 (20)	59 (17)
**Race/ethnicity**		
Non-Hispanic White	527 (80)	321 (85)
Non-Hispanic Black or African American	74 (11)	27 (7)
Asian	37 (6)	9 (2)
Hispanic	27 (4)	8 (2)
American Indian or Alaska Native	14 (2)	6 (2)
Hawaiian or Pacific Islander	6 (1)	2 (1)
Other	14 (2)	6 (2)
**Any public health education^d^**	244 (37)	118 (31)
**Years in public health (mean, SD)^e^**	14.8 (9.4)	16.4 (9.7)
**Public health job position**		
Director, over multiple programs	90 (14)	174 (46)
Program manager	326 (51)	171 (45)
Specialist	214 (33)	22 (6)
Other	11 (2)	8 (2)
**SHD use of the Guide in work unit^f^**		
No	39 (6)	—
Yes, often	273 (44)	—
Yes, sometimes	225 (37)	—
I am not familiar with the Community Guide	78 (13)	—
**LHD use of the Guide across agency for decision making^g^**		
Not used	—	87 (24)
Used in some programs	—	181 (49)
Used consistently across programs	—	17 (5)
Do not know the extent of use of Community Guide in the LHD	—	86 (23)

Abbreviations: — , not applicable; ASTHO, Association of State and Territorial Health Officials; CDC, Centers for Disease Control and Prevention; Guide, The Community Guide; LHD, local health department; NACCHO, National Association of County and City Health Officials; SD, standard deviation; SHD, state health department.

a Values are no. (%) unless otherwise indicated. Not all data sum to total values for n because of missing responses.

b ASTHO-defined state regions.

c SHD participants were asked to select 1 area they worked in from a list of chronic disease areas or indicate that they worked in multiple areas. LHD participants were asked to select all areas they worked in, including diabetes, obesity, physical activity, nutrition, and tobacco.

d Any public health education means that the participant reported having an MPH/MSPH or PhD, DrPH, or ScD in public health.

e Participants were asked to report the overall number of years they had been working in public health.

f SHD participants were asked, “Does your work unit use The CDC Community Guide in its work?” Response options were no, “yes, sometimes,” “yes, always,” and “I am not familiar with The Community Guide.”

g The question about the Guide is from the NACCHO 2016 profile. The survey asked representatives from LHDs, “Which of the following best describes the extent to which The Guide to Community Preventive Services has been used to support or enhance decision making in your LHD over the past 12 months?” The responses included “LHD staff in some programmatic areas have used the Community Guide,” “LHD staff consistently use the Community Guide in all relevant programmatic areas,” “LHD staff have not used the Community Guide,” and “Do not know the extent of use of Community Guide within LHD.”

### Awareness of the Guide

Thirteen percent of SHD participants reported not being aware of the Guide, and 23% of LHD participants reported they did not know the extent of the use of the Guide in the LHD (Table 2 and Table 3). Significant relationships were found between SHD participants reporting awareness of the Guide and position, program area, academic collaboration, and rated importance of forming partnerships. Participants in LHDs who reported that their LHD had an academic collaboration were significantly more likely to have reported having knowledge of use of the Guide in their departments than those that did not report academic collaboration (χ^2^ = 5.3, *P = .*02). The practice of frequently ending programs that should have continued and continuing programs that should have ended were not significantly related to LHD or SHD respondent awareness of the Guide.

**Table 2:** Characteristics of Participants (N = 615) from State Health Departments (SHDs) in the United States that Use and Are Aware of The Community Guide, United States, 2016–2018

Characteristic	State Health Departments					
	Use the Guide^a^			Aware of the Guide^b^		
	Yes (n = 498), n (%)	No (n = 117), n (%)	χ^2^ (*P* Value)	Yes (n = 537) n (%)	No (n = 78) n (%)	χ^2^ (*P* Value)
**Positions^c^**						
Program managers	265 (84)	49 (16)	21.2 (.002)	281 (90)	32 (10)	15.1 (.004)
Specialists	148 (73)	54 (27)		162 (80)	40 (20)	
Directors, overseeing multiple programs	78 (90)	9 (10)		82 (94)	5 (6)	
Other	6 (54.5)	5 (43.5)		10 (91)	1 (9)	
**Program area**						
Obesity	65 (84)	12 (16)	45.5 (<.001)	70 (91)	7 (9)	26.0 (<.001)
Tobacco	62 (94)	4 (6)		62 (94)	4 (6)	
Cancer	77 (89)	9 (11)		78 (91)	8 (9)	
Diabetes	65 (84)	12 (16)		30 (86)	5 (14)	
Cardiovascular	38 (86)	6 (14)		41 (93)	3 (7)	
Multiple areas	140 (84)	26 (16)		150 (90)	16 (10)	
Other areas	88 (62)	53 (38)		106 (75)	35 (25)	
**Jurisdiction size^d^**						
Small (<2.5 million residents)	146 (84)	27 (16)	2.3 (.52)	155 (90)	18 (10)	1.6 (.65)
Medium (2.5–6.4 million)	174 (81)	41 (19)		188 (87)	27 (13)	
Large (>6.4 million)	159 (78)	44 (22)		174 (86)	29 (14)	
**Region^e^**						
New England	80 (79)	21 (18)	12.7 (.01)	86 (85)	15 (15)	7.9 (.09)
South	113 (83)	23 (17)		121 (89)	15 (11)	
West	85 (84)	16 (16)		91 (90)	10 (10)	
Mountains/Midwest	126 (87)	19 (13)		132 (91)	13 (9)	
Mid-Atlantic and Great Lakes	94 (71)	38 (29)		107 (81)	25 (16)	
**Governance of SHDs^f^**						
Shared	64 (74)	23 (26)	6.6 (.04)	72 (83)	15 (17)	4.4 (.65)
Local	307 (80)	75 (20)		331 (87)	51(13)	
State	127 (87)	19 (13)		134 (92)	12 (8)	
**Health department is accredited^g^**						
Yes	341 (83)	71 (17)	2.6 (.10)	363 (88)	49 (12)	0.7 (.40)
No	157 (77)	46 (23)		174 (86)	29 (14)	
**Health department is academic^h^**						
Yes	78 (73)	29 (27)	5.7 (.02)	84 (79)	23 (22)	9.4 (.002)
No or unsure	412 (83)	85 (17)		444 (89)	53 (11)	
**Important for work unit to form partnerships^i^**						
Yes	482 (82)	108 (18)	4.9 (.03)	519 (88)	71 (12)	5.5 (.02)
No	16 (64)	9 (36)		18 (72)	7 (28)	
**Programs that should have continued ended^j^**						
Frequently	40 (83)	8 (17)	0.1 (.76)	44 (92)	4 (8)	0.7 (.42)
Infrequently	450 (82)	102 (19)		484 (92)	68 (12)	
**Programs continue that should have ended^k^**						
Frequently	45 (82)	10 (18)	0.0 (.998)	49 (89)	6 (11)	0.1 (.82)
Infrequently	445 (82)	99 (18)		479 (88)	65 (12)	

Abbreviations: CDC, Centers for Disease Control and Prevention; Guide, The Community Guide; PHAB, Public Health Accreditation Board; SHD, state health department.

a SHD participants were asked, “Does your work unit use The CDC Community Guide in its work?” Response options were no, “yes, sometimes,” “yes, always,” and “I am not familiar with The Community Guide.” “Yes, sometimes” and “yes, always” were coded as “yes, use the Guide”; all other responses were coded as no.

b SHD participants were asked, “Does your work unit use The CDC Community Guide in its work?” Response options were “no,” “yes, sometimes,” “yes, always,” and “I am not familiar with The Community Guide.” “I am not familiar with The Community Guide” was coded as “no, not aware of the Guide”; all other responses were coded as “yes, aware of the Guide.”

c SHD participants self-reported their position. “Specialist” includes epidemiologists and health educators, for example.

d State population in 2012.

e ASTHO-defined state regions.

f Governance type comes from ASTHO classifications.

g Participant’s SHD has PHAB accreditation. Data point is from PHAB.

h This variable comes from the Public Health Foundation. An academic health department is one with a formal affiliation of a health department and an academic institution that trains future health professionals.

i SHD participants were asked to rate the following statement on a scale from 1 (“strongly disagree”) to 5 (“strongly agree”): “It is important for my work unit to develop partnerships with both health and other work sectors to address our state’s health issues.” Strongly agree and agree were coded as yes, and all other responses were coded as no.

j SHD participants were asked to rate the following statement on a scale from 1 (“never”) to 5 (“always”): “How often do ineffective programs, overseen by your work unit, continue when they should have ended?” “Often” and “always” were coded as “frequently,” and all other responses were coded as “infrequently.”

k SHD participants were asked to rate the following statement on a scale from 1 (“never”) to 5 (“always”): “How often do effective programs, overseen by your work unit, end when they should have continued?” “Often” and “always” were coded as “frequently,” and all other responses were coded as “infrequently.”

**Table 3:** Characteristics of Survey Participants (N = 371) From Local Health Departments (LHDs) in the United States That Use and Are Aware of The Community Guide, United States, 2016–2018

Characteristic	Local Health Departments					
	Use the Guide^a^			Aware of Use of the Guide in Their Health Department^b^		
	Yes (n = 198), n (%)	No (n = 173), n (%)	χ^2^ (*P* Value)	Yes (n = 285), n (%)	No (n = 86), n (%)	χ^2^ (*P* Value)
**Jurisdiction size^c^**						
Small (<50,000 residents)	43 (36)	75 (64)	24.8 (<.001)	86 (73)	32 (27)	1.5 (.47)
Medium (50,000–200,000 residents)	69 (54)	58 (46)		100 (79)	27 (21)	
Large (>200,000 residents)	86 (68)	40 (32)		99 (79)	27 (21)	
**Region^d^**						
New England	15 (41)	22 (59)	4.1 (.39)	30 (81)	7 (19)	3.2 (.53)
South	59 (58)	42 (42)		75 (74)	30 (26)	
West	21 (57)	16 (43)		26 (70)	11 (30)	
Mountains/Midwest	32 (49)	33 (51)		48 (74)	17 (26)	
Mid-Atlantic and Great Lakes	71 (54)	60 (46)		106 (81)	25 (29)	
**Governance of LHDs^e^**						
Shared	22 (61)	14 (39)	1.6 (.45)	30 (83)	6 (16.7)	2.6 (.27)
Local	146 (52)	137 (48)		219 (77)	64 (23)	
State	30 (58)	22 (42)		36 (69)	16 (31)	
**Health department is accredited^f^**						
Yes	73 (65)	40 (32)	8.4 (.004)	89 (79)	24 (21)	0.4 (.55)
No	124 (48)	133 (52)		195 (76)	62 (24)	
**Health department is academic^g^**						
Yes	152 (57)	117 (44)	5.8 (.02)	214 (80)	55 (20)	5.3 (.02)
No or unsure	41 (42)	56 (58)		66 (68)	31 (32)	
**Important for work unit to form partnerships^h^**						
Yes	174 (54)	151 (47)	0.1 (.76)	253 (78)	72 (22)	1.8 (.18)
No	23 (51)	22 (49)		31 (69)	14 (31)	
**Programs that should have continued ended^i^**						
Frequently	56 (54)	47 (46)	0.0 (>.99)	83 (81)	20 (19)	0.9 (.34)
Infrequently	133 (54)	112 (46)		186 (76)	59 (24)	
**Programs continue that should have ended^j^**						
Frequently	33 (58)	24 (42)	0.4 (.53)	45 (79)	12 (21)	0.1 (.73)
Infrequently	152 (53)	133 (47)		219 (77)	66 (23)	

Abbreviations: Guide, The Community Guide; LHD, local health department; NACCHO, National Association of County and City Health Officials; PHAB, Public Health Accreditation Board.

a The question about the Guide is from the NACCHO 2016 Profile. The survey asked representatives from LHDs, “Which of the following best describes the extent to which The Guide to Community Preventive Services has been used to support or enhance decision making in your LHD over the past 12 months?” The responses included “LHD staff in some programmatic areas have used the Community Guide,” “LHD staff consistently use The Community Guide in all relevant programmatic areas,” “LHD staff have not used The Community Guide,” and “Do not know the extent of use of Community Guide within LHD.” The first 2 responses were coded as “yes, use the Guide” and the other 2 responses were coded as no.

b The question about the Guide is from the NACCHO 2016 Profile. The survey asked representatives from LHDs, “Which of the following best describes the extent to which The Guide to Community Preventive Services has been used to support or enhance decision making in your LHD over the past 12 months?” The responses included “LHD staff in some programmatic areas have used The Community Guide,” “LHD staff consistently use The Community Guide in all relevant programmatic areas,” “LHD staff have not used The Community Guide,” and “Do not know the extent of use of Community Guide within LHD.” The last response was coded as “no, not aware of the use of the Guide” and the other 3 responses were coded as “yes, aware of the use of the Guide.”

c Population from NACCHO 2016 Profile.

d ASTHO-defined state regions.

e From NACCHO 2016 Profile.

f LHD participants were asked, “Is your health department accredited or preparing to apply for accreditation through the Public Health Accreditation Board (PHAB)?” Participants could select from responses “We are currently accredited,” “Yes, and we have recently applied but are not yet accredited,” “Yes, but we have not yet applied,” no, or “Unsure.” “We are currently accredited” was coded as yes, and all other responses were coded as no.

g LHD participants were asked, “Does your agency currently participate in any academic partnerships (arrangement between an academic institution and a governmental public health agency that provides mutual benefits in teaching, research, and service)?” Participants could respond yes, no, or “unsure.”

h LHD participants were asked how much they agree with the statements, on a scale of 1 to 7, from strongly disagree to strongly agree, “It is important to my agency to have partners in health care to address population health issues” and “It is important to my agency to have partners in other sectors (outside of health) to address population health issues.” If they indicated on one or both of the items a 6 or 7, then the item was coded as yes. If the participant did not select a 6 or 7 for either question, the item was coded as no.

i LHD participants were asked “In your opinion, how often do programs end that should have continued (ie, end without being warranted)?” They could select “never,” “rarely,” “sometimes,” “often,” “always,” “I do not know,” or “not applicable.” “Often” and “always” were coded as “frequently,” and all other options were coded as “infrequently.”

j LHD participants were asked, “In your opinion, how often do programs continue that should have ended (ie, continue without being warranted)?” They could select “never,” “rarely,” “sometimes,” “often,” “always,” “I do not know,” or “not applicable.” “Often” and “always” were coded as “frequently,” and all other options were coded as “infrequently.”

### SHD use of the Guide

Eighty-one percent of SHD participants reported using the Guide in their work (Table 1). We found a significant relationship between SHD region and use of the Guide (χ^2^ *= *12.7, *P = .*01 (Table 2). Participants in the Mountains/Midwest region were more likely than those in other regions to report using the Guide (87%). We also found a significant relationship between program area and use of the Guide (χ^2^ *= *45.5, *P < .*001). Those working in tobacco (94%) were most likely to report using the Guide in their work. We found a significant relationship between respondent-rated importance of forming partnerships and the Guide use in SHDs (χ^2^ *= *4.9, *P = .*03). Participants that rated forming partnerships as important were more likely than those who did not to use the Guide (82% vs 64%). Participants who reported their SHD collaborated with academic institutions were significantly less likely than participants who reported no collaboration with academic institutions to use the Guide (χ^2^ *= *5.8, *P = .*02). Working at an accredited SHD was not significantly related to SHD participants using the Guide (χ^2^ *= *2.6, *P = .*10). We found no significant relationship between SHD participants that reported their work units frequently ended programs that should have continued or frequently continued programs that should have ended and use of the Guide. Of SHD participants who reported their work unit frequently ended programs that should have continued, 83% reported that they used the Guide compared with 82% use of the Guide among those who reported that these programs ended infrequently (χ^2^ = 0.1, *P = .*76). Among SHD participants that reported their work units both frequently and infrequently continued programs that should have ended, 82% also reported using the Guide (χ^2^ = 0.0, *P = *>*.*99).

### LHD use of the Guide

Fifty-four percent of LHD participants reported using the Guide in their work (Table 1). Participants at LHDs with academic collaborations were more likely to use the Guide than those reporting no academic collaboration (Table 3). LHD participants in jurisdictions with fewer than 50,000 residents were less likely to report use of the Guide (36%) than those in medium (54%) or large (68%) jurisdictions (χ^2^ *= *24.8, *P < .*001). Those working at an accredited LHD were significantly more likely to report use of the Guide than participants working at nonaccredited LHDs (χ^2^ *= *8.4, *P = .*004). Among LHD participants, we did not find a significant relationship between use of the Guide and perceived frequency of ending programs that should have continued or continuing programs that should have ended. Among LHD participants that reported their work units frequently and infrequently ended programs that should have continued, 54% also reported using the Guide (χ^2^ *= *0.0, *P* > .99). Of LHD participants who reported their work unit frequently continued programs that should have ended, 58% reported that they used the Guide compared with 53% who reported that these programs continued infrequently (χ^2^ = 0.4, *P = .*53).

## Discussion

We found that SHDs and LHDs are using the Guide in their work and that several factors are related to its awareness and use. In SHDs, state governance of LHDs and perceived importance of partnerships were positively related to respondent use, and position was related to both respondent awareness and use of the Guide. In LHDs, large jurisdiction size, accreditation, and academic collaboration were positively related to participants reporting agency use of the Guide. Inappropriate continuation or inappropriate termination of programs was not significantly related to use or awareness of the Guide in SHDs or LHDs.

A larger percentage of SHD than LHD participants reported using the Guide in their work. Approximately 80% of SHD and 54% of LHD participants reported that they or their agency use the Guide in their work. However, 13% of SHD participants reported they were unaware of the Guide, and 23% of LHD participants reported that they do not know the extent of use of the Guide in their department. The rates of use among LHDs are higher than those found in previous studies. Lovelace et al, using data from the NACCHO 2010 Profile, found that only 22% of LHDs used the Guide in their work (13). It could be that increased awareness of evidence-based public health has led to more LHDs reporting using the Guide in recent years. In the 2016 NACCHO Profile, 37% of the 1,890 LHDs that responded reported Guide use (11). The higher rate of use that we found could reflect the selection of LHDs included in the 2017 LHD survey, as it includes only LHDs that implement community health programs for chronic disease prevention. Hannon et al found that only about half of cancer control practitioners they surveyed had ever used an evidence-based practice resource such as the Guide (14). Brownson et al found that 90% of SHD participants were aware of the Guide, which was similar to our finding that 87% of SHD participants were aware (15). The high rates of use and awareness of the Guide that we found suggests that health departments are using the Guide and are interested in learning about the effectiveness of EBIs.

We found a significant relationship between respondent ratings of importance of forming partnerships with awareness and use of the Guide in SHDs but not in LHDs. The lack of the significant relationship in LHDs may be because of the lack of variance in response, as 88% of respondents rated forming relationships as important. This relationship between use and awareness of the Guide with the importance of forming partnerships may exist because many of the EBIs provided in the Guide call for or include a partnership component (9). It may also be that health departments with partnerships have higher capacity to implement EBIs and use the Guide in their work. Some studies found that a health department having higher capacity in administrative evidence-based practices is related to the department having stronger partnerships (16), while others found no difference in higher-capacity versus lower-capacity health departments (17). In either case, the need for stronger partnerships between public health and primary care has been recommended by CDC (18). In addition to collaborating with health care, public health can leverage transdisciplinary partnerships with other partners to address public health problems that have complex and various causes.

SHD participants that reported collaborating with academic institutions were significantly less likely to be aware of or to use the Guide in their work, whereas those in LHDs with academic collaborations were more likely to use the Guide and to be aware of its use in their department. Studies have found that LHDs engaged in academic collaborations were more likely to report supports for the use of evidence-based decision making and more likely to implement EBIs than LHDs not engaged in such partnerships (19). Recent studies demonstrated successful formal and informal collaborations between academic institutions and SHDs or LHDs (14,19). Like partnerships, academic collaborations may allow health departments to fill gaps in expertise and resources that the departments themselves may not have. It may be that academic collaboration is positively related to LHD use of the Guide because the increased capacity that these collaborations offer make LHDs more interested in EBIs or more confident to implement them in their work. SHD academic partners, rather than the SHD itself, could be using the Guide. It could also be that academic partners support the use of other evidence-based resources similar to the Guide, such as Cochrane Public Health and Health Evidence (20,21).

Use of the Guide is related to population size in LHDs but not in SHDs. Other studies have found a relationship between LHD population size and performance (22). No studies have examined LHD population size and use of the Guide; however, Harris et al found that LHD public health practitioners who worked in larger population size jurisdictions were more likely to adapt evidence-based public health practices (23). LHDs serving larger population sizes may have more access to well-trained staff and other community partner expertise and resources, such as hospitals and universities (13). Additionally, it could be that EBIs are more difficult to adapt to smaller or rural populations. Dodson et al and Jacob et al found a lack of information and training of public health practitioners on how to adapt EBIs to different populations and settings (24,25). It may be that smaller LHDs are less likely to use the Guide because they feel the EBIs do not apply to their populations or that they do not know how to adapt EBIs to fit their population. Training public health practitioners on adapting EBIs to different populations and settings is vital to making the most out of the information provided in the Guide.

The Guide may be used less frequently by some participants because many SHDs and LHDs are funded by federal or state agencies who provide implementation guides showing which interventions are allowable uses of award monies. In one qualitative interview study, SHD chronic disease practitioners reported that funding drives much of EBI selection and that often funding is tied to using particular EBIs (26). For example, CDC’s National Center for Chronic Disease Prevention and Health Promotion provides funding to states to prevent and manage diabetes, heart disease, and stroke and provides guidelines for which EBIs SHDs can use with the funding (27).

Accreditation is significantly related to LHDs using the Guide but not significantly related to SHDs using it. The Public Health Accreditation Board (PHAB), a voluntary accreditation board for public health departments, requires that LHDs and SHDs use evidence in their decision making and programs (28). PHAB lists the Guide as an evidence-based resource (9). Studies have found that accreditation is related to use of more EBIs in LHDs (29) and use of evidence in SHDs (30). In a study by Cilenti et al, LHD participants interviewed reported that accreditation helped to promote use of evidence-based practices, because it required implementing approaches that resulted in desired outcomes (31).

Mis-implementation and inappropriate continuation or termination of programs were not significantly related to use of, awareness of, or awareness of use of the Guide in SHD or LHDs. It is likely that the information provided in the Guide is not enough for SHDs and LHDs to successfully adopt only EBIs or discontinue programs that are not EBIs because of the many barriers related to adopting EBIs in health departments (24). According to findings from Brownson et al and Hannon et al nearly all participants reported that they desired more training to enhance the use of evidence-based public health resources like the Guide (14,15). Information alone on EBIs from resources like the Guide does not seem to be enough to prevent mis-implementation of public health programs.

Our study and related literature provide a set of actionable steps for health departments and partner organizations that can enhance the uptake of EBIs in the Guide:

- Encourage health departments to post Guide links on their intranet and on their public-facing websites.
- Encourage health departments to include the Guide link in their internal documents, such as requests for proposals to organizations they in turn fund, program plans, evaluation plans, and internal review documents for new programs.
- Conduct focused workshops and provide technical assistance to raise awareness of the Guide and build skills for EBI implementation (32).
- Use diffusion theory to tailor implementation strategies (eg, assess feasibility and cost, describe the advantages over existing practices) to increase use of the Guide (33).
- Conduct qualitative data collection to better understand how best to tailor Guide findings to settings and populations being served by a particular health department.
- Make use of Guide findings in health department accreditation efforts (34).
- Encourage funders to promote the Guide and implementation tools in requests for proposals targeted to health departments.

Our study has limitations. First, the LHD survey did not ask about the Guide use in the same way that the SHD survey did. This required using the question from the 2016 NACCHO Profile. Second, the LHD survey had only one respondent from each health department, whereas the SHD survey had multiple responses from the same health department. This difference affects interpretation of the findings, because LHDs were asked for their perspectives about their entire department, while SHD participants were asked their perspectives about their work units. Third, the questions for LHDs and SHDs were not exactly the same. Direct comparisons between LHDs and SHDs should not be made; instead, SHD factors should be compared with other SHDs and LHDs to LHDs. Fourth, all data were self-reported. Participants may not know everything about the health department they work in; thus, some information reported could be inaccurate. Fifth, data in the analysis were used from surveys that were conducted in different years, so temporal factors could have played a role in some differences between surveys.

The Guide can help make public health practitioners more aware of EBIs (33). As LHD practitioners appear to use the Guide less than do SHD practitioners, an opportunity exists to increase the use of the Guide and potentially increase uptake of EBIs with promotion of the Guide. Our study supports findings of other researchers that partnerships, training on adapting EBIs to different settings and populations, and resources are likely needed to successfully apply information on EBIs from the Guide.

## Post-Test Information

To obtain credit, you should first read the journal article. After reading the article, you should be able to answer the following, related, multiple-choice questions. To complete the questions (with a minimum 75% passing score) and earn continuing medical education (CME) credit, please go to http://www.medscape.org/journal/pcd. Credit cannot be obtained for tests completed on paper, although you may use the worksheet below to keep a record of your answers.

You must be a registered user on http://www.medscape.org. If you are not registered on http://www.medscape.org, please click on the “Register” link on the right hand side of the website.

Only one answer is correct for each question. Once you successfully answer all post-test questions, you will be able to view and/or print your certificate. For questions regarding this activity, contact the accredited provider, CME@medscape.net. For technical assistance, contact CME@medscape.net. American Medical Association’s Physician’s Recognition Award (AMA PRA) credits are accepted in the US as evidence of participation in CME activities. For further information on this award, please go to https://www.ama-assn.org. The AMA has determined that physicians not licensed in the US who participate in this CME activity are eligible for AMA PRA Category 1 Credits™. Through agreements that the AMA has made with agencies in some countries, AMA PRA credit may be acceptable as evidence of participation in CME activities. If you are not licensed in the US, please complete the questions online, print the AMA PRA CME credit certificate, and present it to your national medical association for review.

## Post-Test Questions

### Study Title: Use and Awareness of The Community Guide in State and Local Health Department Chronic Disease Programs

#### CME Questions

1. You are advising a local health department (LHD) about implementation of evidence-based interventions (EBIs). According to the pooled analysis of self-report data from recent national surveys by Rodriguez Weno and colleagues, which of the following statements about prevalence of and characteristics related to use and awareness of the Community Guide (Guide) by LHDs is correct?
  - Only one-third of LHD respondents reported that their agency uses the Guide
  - Participants in LHDs with vs without an academic collaboration were more likely to have reported having knowledge of Guide use in their departments
  - LHD participants in smaller jurisdictions were most likely to report use of the Guide
  - Respondents working at an accredited LHD were significantly less likely than respondents working at nonaccredited LHDs to report Guide use
2. According to the pooled analysis of self-report data from recent national surveys by Rodriguez Weno and colleagues, which of the following statements about prevalence of and characteristics related to use and awareness of the Guide by state health departments (SHDs) is correct?
  - 81% of SHD respondents reported that their agency uses the Guide, but 13% reported not being aware of the Guide
  - Respondents in the Mountains/Midwest region were less likely than respondents in other regions to report using the Guide
  - Only 56% of respondents working in the tobacco industry reported Guide use in their work
  - Respondent-rated importance of forming partnerships was not significantly associated with Guide use
3. According to the pooled analysis of self-report data from recent national surveys by Rodriguez Weno and colleagues, which of the following statements about public health implications of prevalence of and characteristics related to use and awareness of the Guide by LHDs and SHDs is correct?
  - Use of the Guide alone is sufficient for health departments to implement EBIs
  - Guide use currently appears to be optimal among LHDs and SHDs
  - Suggested actionable steps for health departments and partner organizations are unlikely to enhance uptake of EBIs in the Guide
  - Organizational-level changes (eg, sharing information about the Guide and providing training on how to best use it) may increase its awareness and use
